# Evaluation of graphene/crosslinked polyethylene for potential high voltage direct current cable insulation applications

**DOI:** 10.1038/s41598-021-97328-x

**Published:** 2021-09-13

**Authors:** Yuan Li, Guangya Zhu, Kai Zhou, Pengfei Meng, Guodong Wang

**Affiliations:** College of Electrical Engineering, Sichuan University, Chengdu, 610065 China

**Keywords:** Electrical and electronic engineering, Plasma physics

## Abstract

This paper evaluates the potential usage of graphene/crosslinked polyethylene (graphene/XLPE) as the insulating material for high voltage direct current (HVDC) cables. Thermal, mechanical and electrical properties of blends with/without graphene were evaluated by differential scanning calorimetry (DSC), tensile strength, DC conductivity, space charge measurements and water tree aging test. The results indicate that 0.007–0.008% weight amount of graphene can improve the mechanical and electrical insulation properties of XLPE blends, namely higher tensile/yield strength, improved space charge distribution, and shorter/fewer water tree branches. The improvements mainly attribute to the high stiffness of graphene, deep traps introduced by the interaction zones of graphene and XLPE, and the blockage effect of graphene within XLPE. For thermal performance of XLPE blends, graphene nano-fillers have but limited improvement. The crystallinity of the blends barely changes with the addition of graphene. However, the crosslinking degree increases as the additive-like amounts of graphene doped. The above findings provide a guide for tailoring lightweight XLPE materials with excellent mechanical and electrical performances by doping them with a small amount of graphene.

## Introduction

High voltage direct current (HVDC, ≥ 1.5 kV) transmission possesses a good number of advantages such as long transmission distance, high transmission efficiency and low active power loss. Along with the mass construction of the power grid, the size as well as the voltage grade of HVDC transmission increases synchronously^1–3^. As one of the most important part of HVDC, cables directly determine the safety of the system. For cables in alternating current (AC) transmission system, crosslinked polyethylene (XLPE) has been widely used as their insulating material due to its high breakdown strength, low dielectric loss, good mechanical properties and improved thermal resistance^4–6^. However, the application of XLPE in high voltage direct current (HVDC) cables still faces multiple problems, the most serious of which is the space charge accumulation and its corresponding consequences, namely electric field distortion, partial discharge, or even breakdown^7–9^. To improve the safety and stability of cables in HVDC transmission, it is necessary to modify XLPE materials so as to suppress the accumulation of space charges. Moreover, a quantum leap is also needed for the next generation polymeric HVDC cables with a further increased transmission voltage up to 800 kV^10^.

Up to now the most practical strategy to improve the space charge resistance of XLPE is by nano doping^11^. 1–100 nm nanoparticles are uniformly dispersed into the polymer, through which both the physical and chemical properties of the polymer are modified, mostly improved^12,13^. The doped polymer, if used as dielectrics, is hence called nano-dielectrics. Lewis first studied nano-dielectrics in 1994^14^. Since then the concept began to gain more research attention. e.g., Smith studied the effect of metal oxide nanoparticle fillers on the electrical properties of XLPE samples and presented the hypothesis, that the nanoparticles will buildup homo-charge at the electrodes, which increases the voltage required for space charge injection due to blocking by the homo-charge^15^. Wang studied the effect of nano-TiO_2_ on DC XLPE cables, and found that adding a small amount of nano-TiO_2_ to XLPE can improve the dielectric properties of polymers, including the crystallinity, conductivity activation energy and DC breakdown strength^16^. Nevertheless, it is also pointed out that inorganic nanoparticles may cause particle agglomeration and thus being unable to guarantee improvements to the overall electrical properties of the nano-dielectrics^17^. Furthermore, high loading of inorganic nanoparticle will also make the dielectric heavier, which restrict its industrial application^18^. Dielectrics with less nanoparticle load and better electrical performance is therefore highly desired.

Graphene, with its typical close packed two-dimensional structure and unique properties, has been used in various areas, including transistor, hydrogen storage and photovoltaics technologies^19–24^. Recent studies on graphene also indicated it being a promising nanoparticle filler to improve the electrical and thermal properties of dielectrics, though its effect on the mechanical properties has not been investigated yet^25,26^.

In this paper, graphene/XLPE nano-dielectric samples with graphene contents of 0%, 0.002%, 0.004%, 0.006%, 0.008%, 0.01% were prepared by solution blending method. Based on measurements of various parameters including thermal performance, tensile strength, DC conductivity and space charge distribution, the effect of nano-graphene on thermal, mechanical and electrical properties of XLPE was thoroughly studied. Meanwhile, the nano-dielectric samples were subjected to accelerated water tree aging test to explore the potential inhibition effect of nanographene on water tree aging process of XLPE. We believe that nanographene can decrease the growth rate of water tree both by increasing the yield strength of XLPE and hindering water migration within it.

## Results and discussion

### Thermal performance

**Figure 1:**
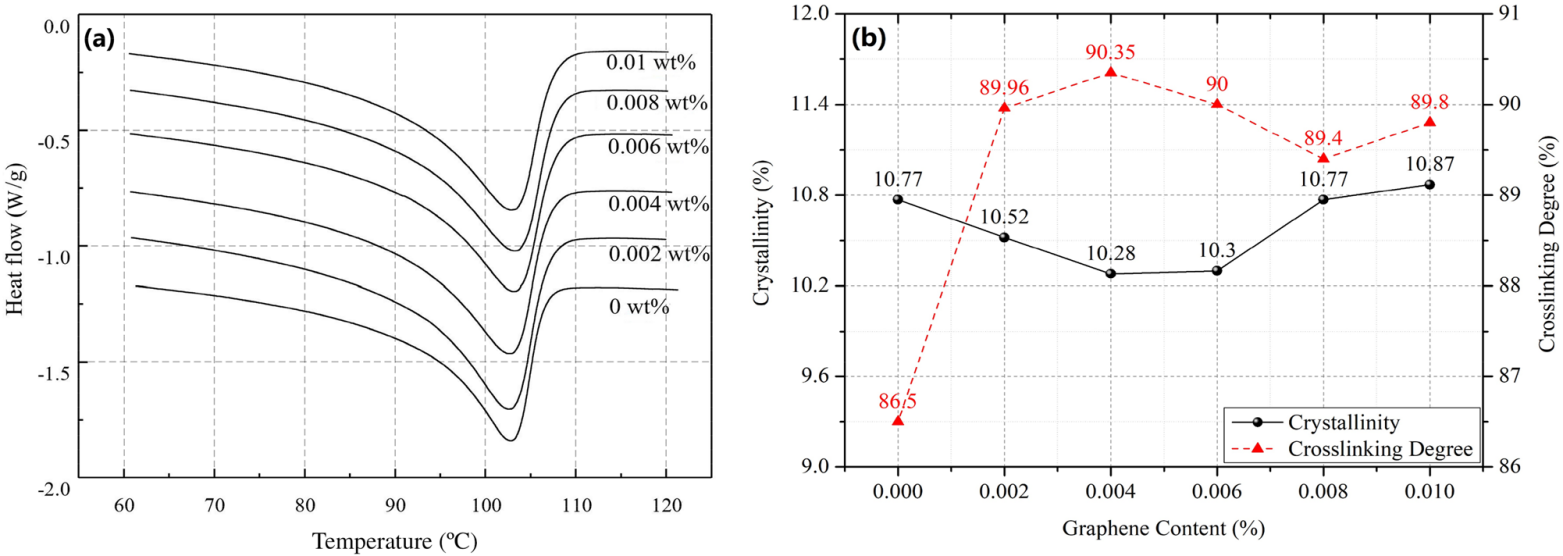
(**a**) DSC curves of graphene/XLPE samples (0%, 0.002%, 0.004%, 0.006%, 0.008% and 0.01%). (**b**) Crystallinity and crosslinking degree of graphene/XLPE samples (0%, 0.002%, 0.004%, 0.006%, 0.008% and 0.01%).

The obtained differential scanning calorimetry (DSC) curves of graphene/XLPE samples (0%, 0.002%, 0.004%, 0.006%, 0.008% and 0.01%) are shown in Fig. 1a. Based on the results, the crystallinity of the samples is calculated and plotted in Fig. 1b, together with the crosslinking degree of the samples measured by gel method^27^.

In Fig. 1a, it can be seen that the DSC curves of the samples remain largely the same as the addition of graphene increases. The melting peaks of the curves show little changes, being around 103 °C. This trend is also illustrated in Fig. 1b, as the crystallinity of the samples remains stable with more graphene doped. However, changes in the crosslinking degree indicate that the inclusion of graphene will increase the thermal stability of XLPE samples. For samples without graphene, the average crosslinking degree is 86.5%, nearly 3.5% lower than that of the graphene-doped samples. Therefore, it is concluded that the addition of graphene has but limited improvement for the thermal performance of XLPE blends, mainly on crosslinking degree.

### Tensile strength

**Figure 2:**
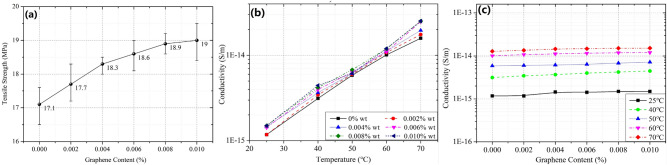
(**a**) Tensile strength of graphene/XLPE samples (0%, 0.002%, 0.004%, 0.006%, 0.008% and 0.01%). (**b**) and (**c**) DC conductivity of graphene/XLPE samples under different temperatures (25 °C, 40 °C, 50 °C, 60 °C, 70 °C): (**b**) temperature as the X-axis, (**c**) Graphene content as the X-axis.

The tensile strength of graphene/XLPE samples (0%, 0.002%, 0.004%, 0.006%, 0.008% and 0.01%) are shown in Fig. 2a, where an obvious increment can be observed as the addition of graphene increases. For pure XLPE sample, its average tensile strength is 17.1 MPa. When 0.002 wt%, 0.004 wt%, 0.006 wt% and 0.008 wt% graphene are doped, the strength changes to 17.7 Mpa, 18.3 MPa, 18.6 MPa, and 18.9 MPa, respectively. On the other hand, as the graphene content continues to increase, the tensile strength gradually tends to a certain value, also seen from the flat tail of the curve in Fig. 2a.

The enhancement in tensile strength of graphene-doped XLPE samples can be attributed to the following factors: (1) the addition of graphene nanoparticles improves the microstructure of the XLPE through minimization of the size of voids/defects inside the sample, and improves the alignment of structural elements of the microstructure at all stages of XLPE sample manufacturing; and (2) the graphene serves as a reinforcing phase to further improve the mechanical properties^28,29^. Atomistic ReaxFF and large-scale molecular dynamics simulations by Gao et al. also elucidate the ability of graphene to modify the microstructure of polymers by promoting favorable edge chemistry and polymer chain alignment^30^.

### DC conductivity

Figure 2b, c shows the DC conductivity of graphene/XLPE samples (0%, 0.002%, 0.004%, 0.006%, 0.008% and 0.01%) under different temperature. In Fig. 2b, the conductivity increases exponentially as the temperature rises, the trend of which has been observed and well explained by many other researchers^31–33^. In Fig. 2c, however, the change of conductivity with graphene content barely happens. Note that graphene possesses a high conductivity, this insignificant change is unexpected.

To explain the phenomenon, the percolation theory needs to be considered: when the graphene content is 0.01%, the fillers are well-dispersed and the nearest distance between neighbor fillers can be as large as tens micrometers, indicating the filler content is much lower than the percolation threshold^34^. Therefore, it is speculated that no conducting path of charge carriers forms, resulting in the consistency of the conductivity.

### Space charge distribution

**Figure 3:**
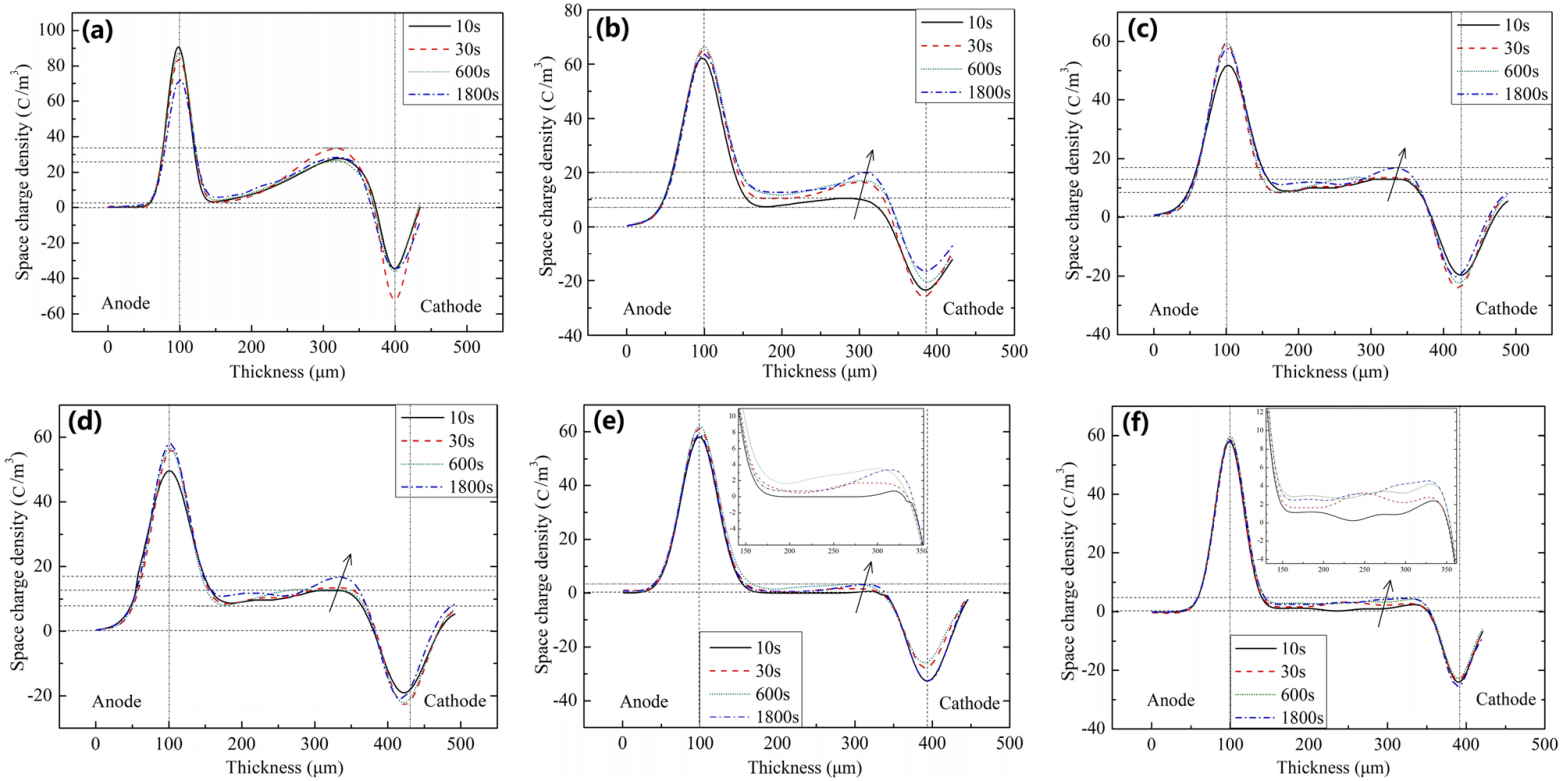
Space charge distribution of graphene/XLPE samples: (**a**) XLPE, (**b**) XLPE + 0.002 wt% graphene, (**c**) XLPE + 0.004 wt% graphene, (**d**) XLPE + 0.006 wt% graphene, (**e**) XLPE + 0.008 wt% graphene, (**f**) XLPE + 0.01 wt% graphene.

Based on PEA method described in the method section, the space charge distribution of graphene/XLPE samples (0%, 0.002%, 0.004%, 0.006%, 0.008% and 0.01%) was measured, the results of which are shown in Fig. 3.

For pure XLPE samples, hetero-charges accumulated dramatically near the cathode, where only a few homo-charges appeared near the anode. Meanwhile, the density of hetero-charges increased obviously with the polarization time, whereas the density of homo-charges showed little changes.

For graphene/XLPE samples, however, fewer hetero-charges accumulated near the cathode compared with pure XLPE samples. The density of hetero-charges decreased significantly with increasing graphene content, and reached the minimum at the graphene content 0.008%. In addition, the density of homo-charges in the samples also decreased gradually with the increase of the graphene content and reached the minimum value at the same graphene content, namely 0.008%.

The above phenomena show that the density of homo-charges in graphene /XLPE samples is higher than that of the pure XLPE samples. One possible reason is that the graphene-polymer interaction zones will introduce deep traps between graphene and PE, thus suppressing the charge carrier transport, leading to the accumulation of large quantities of homo-charges in the vicinity of both electrodes^35^. This explains the trend of space charge density when the graphene content is lower than 0.008%. On the other hand, as the graphene content continues to increase, the overlapping sites of the graphene-polymer interaction zones would provide low-resistance paths for electrons between molecular chains, thus accelerating the transport of electrons through chain barriers, and leading to significant amount of space charge accumulation in the bulk of polymer^36^. Therefore, it can be concluded that appropriate amount of graphene nano-particles can improve the space charge distribution with XLPE samples.

### Water tree aging characteristics

Water trees are the dendritic paths formed in a wide range of hydrophobic polymeric insulating materials when exposed to electric stress and water immersion^37^. It is regarded as one of the principal aging factors of XLPE cables, as its formation in the cable will cause the reduction in breakdown voltage of cable’s insulating layer^38,39^. As a result, in the development of graphene/XLPE nano-dielectric which serves as the insulating materials of power cables, the water tree aging characteristics should also be considered.

For this purpose, pure XLPE samples and 0.007 wt% graphene/XLPE samples were prepared and subjected to water tree aging tests, respectively. The test platform was shown in Fig. 7, where 1.7 mol/L NaCl solution was used to initiate water trees at the 1.5 mm deep pinhole tips embedded on the surface of the sample. The test voltage was set to 7.5 kVrms with a frequency of 400 Hz. The water tree aging timespan was set to 30 days. After the test, the aged samples were first sliced by a YD-2508 slicer, and then dyed by methylene blue for further microscopic observations.

**Figure 4:**
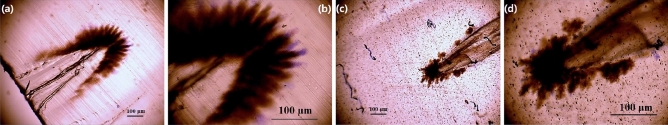
(**a**) and (**b**) Water tree morphology in pure XLPE samples. (**c**) and (**d**) Water tree morphology in 0.0007 wt% graphene / XLPE samples.

Figure 4 shows the microscopic images of water trees in pure XLPE slice and 0.007 wt% graphene/XLPE slice, respectively. The magnification was 64 times for Fig. 4a, c, and 160 times for Fig. 4b, d. The results are as follows:

In Fig. 4a, b, the water tree branches are relatively dispersed, where the longer branches are distributed at the tip of the pinhole. Meanwhile, more than one-third of the pinhole tip surface is covered by branches with a radial growth trend, and the growth direction is basically consistent with the direction of the electric field. Compared with Fig. 4a, b, the number of water tree branches in Fig. 4c, d is significantly fewer. Although a large number of branches concentrates in the small area of the pinhole tip, branches in other areas of the tip are sparse. Besides, the growth direction of water tree branches in graphene/XLPE samples is relatively random. The whole shape of the water tree is more irregular than that in pure XLPE samples.

To better quantify the results of the water tree aging test, we measured the length of the water tree branches in pure XLPE slice and 0.007 wt% graphene/XLPE slice, and calculated their average value respectively. The results indicated that the average tree length in 0.007 wt% graphene/XLPE slice is 85.6 μm, significantly (16.2 μm/15.9%) shorter than 101.8 μm in pure XLPE slice. All the above experimental results indicate that graphene nanoparticles can inhibit the growth of water trees in XLPE effectively.

To explain the inhibiting effect of graphene nanoparticles, we need to focus on the initiation and growth processes of water trees, which according to the known research, are caused by the fatigue of the material. The fatigue, meanwhile, is mainly attributed to two factors-electric stress, and chemical corrosion^40,41^.

**Figure 5:**
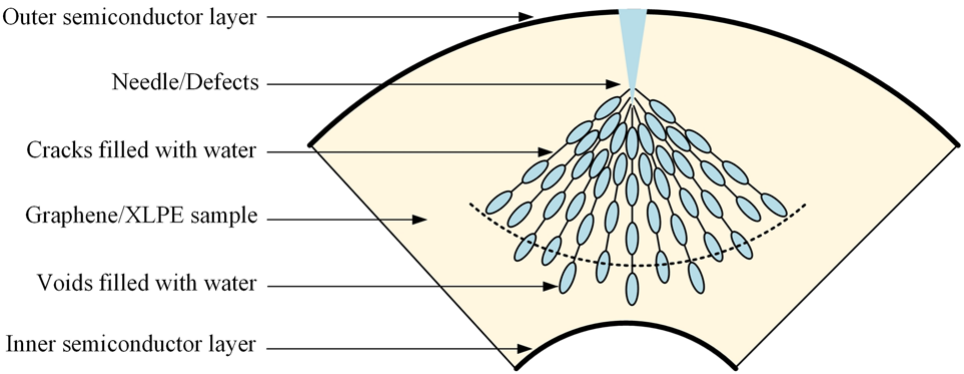
Void-crack model of water trees in insulating polymer.

Electric stress induces water tree by the followings. Once water immerse the insulating material, it will deform along the direction of the electric field force, during which its shape changes from spherical to ellipsoidal. The deformed water droplets will apply the extrusion force to the material. When the pressure exceeds the tensile stress of the material, the molecular chain of the latter will break, leading to the generation of sub-micro cavities (diameter about 0.1–5 μm) and micro channels (diameter about 100 nm), as shown in Fig. 5. Subsequently, water will fill the cavities and the channels. The above process occurs repeatedly, resulting in the formation of water trees.

On the other hand, the water tree induced by chemical corrosion mainly considers the effects of oxidation and ion electroosmosis. When water immerses the material, oxide is also introduced. The oxidation of the polymer will then happen on the wall of some isolated micro-voids, which eventually produce sub-micro cavities in the amorphous region of the material. Meanwhile, ions dissolved in the water will also migrate and cause electroosmosis effect within the material, resulting in the formation of micro channels. These channels, together with the cavities produced by the oxidation process, form the water trees within the insulating materials.

**Figure 6:**
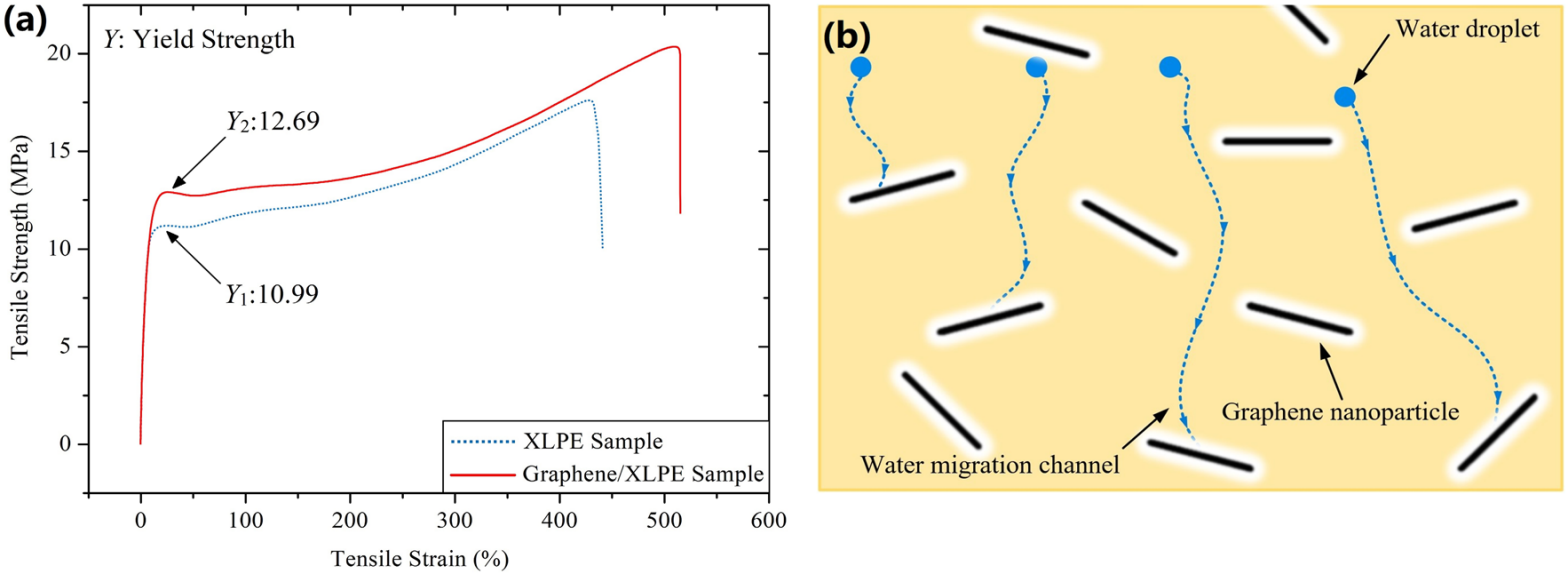
(**a**) Mechanical properties of pure XLPE sample and 0.007 wt% graphene/XLPE sample. (**b**) Illustration of the blockage effect of graphene nanoparticles on water migration in XLPE.

Now we consider the effect of graphene nanoparticles on the above two processes. Figure 6a shows the mechanical properties of pure XLPE sample and 0.007 wt% graphene/XLPE sample. It is quite obvious that graphene nanoparticles enhance the yield strength of XLPE by 5.5%, from 10.99 MPa to 12.69 MPa. This is predictable hence embedding materials with high stiffness into polymers leads to an increase in material stiffness, allowing for stress redistribution from a low modulus matrix to a high modulus filler phase^42^. Since the electric stress-induced water tree is closely related to the yield strength of the material, it is less likely that water trees grow in graphene/XLPE samples.

Meanwhile, the hydrophobic graphene inhibits the growth of water tree also by hindering the water migration within XLPE sample. As illustrated in Fig. 6b, when there is no graphene dopped in XLPE samples, the moisture will migrate along the inherent cracks within the sample, carrying oxide and ions with it. However, when enough graphene nanoparticles are dopped, the inherent cracks of XLPE will be blocked by graphene, which makes it difficult for water to move forward, thus weakening the transportation of oxide and ions. The initiation and growth of water trees in graphene/XLPE samples is eventually inhibited.

## Conclusion

In conclusion, we have fabricated graphene/XLPE nano-dielectric samples by a solution method. The effect of graphene addition on the thermal performance, tensile strength, DC conductivity, space charge distribution and water tree aging behavior of graphene/XLPE composites were investigated. Results indicate that XLPE filled with graphene nanoparticles exhibited excellent mechanical and electrical insulation properties, namely higher tensile/yield strength, improved space charge distribution, and shorter water tree branches. The improvements mainly attribute to the high stiffness of graphene, deep traps introduced by interface between graphene and XLPE, and the blockage effect of graphene within XLPE. In addition, it was found that graphene nano-fillers have but limited improvement for the thermal performance of XLPE blends, mainly on crosslinking degree. The above findings provide a guide for tailoring lightweight XLPE materials with excellent mechanical and electrical performances by doping them with a small amount of graphene. The composite proves its potential for UHV HVDC cable insulation applications.

## Methods

### Sample preparation

Polyethylene (PE) with a density of 0.98 g/cm^3^ was purchase from Kingfa Science & Technology Co., Ltd. Graphene nanoplatelets with properties shown in Table 1 were supplied by Ashine Graphene Co., Ltd.. The nano-dielectric samples were prepared as follows: (1) Vacuum dry the graphene for 24 h prior to compounding. (2) Mix the graphene of certain mass with xylene, and sonicate the mixture for 1 h. (3) Transfer the mixture to a flask with certain amount of PE and xylene, and heat the mixture in an oil bath (100 °C) for 12 h under high-speed stirring. (4) Wash the mixture with ethyl alcohol and vacuum dry it at 80 °C for 24 h. (5) Add the cross-linking agent to the dried mixture and crosslink it at 160 °C/15 MPa for 30 min. (6) Degas the crosslinked product for 24 h to remove by-products.

**Table 1:** Properties of graphene nanoplatelets adopted in the experiment.

Properties	Value
Appearance	Black powder
Purity	> 98 wt%
Thickness	0.55–3.75 nm
Equivalent diameter	0.5–3 μm
Surface area	450–500 m^2^/g
Layers	< 10
Conductivity	≈2 × 10^4^ S/m

Based on the above procedures, we prepared graphene/XLPE nano-dielectric samples with graphene contents by weight of 0%, 0.002%, 0.004%, 0.006%, 0.008% and 0.01%, respectively. The samples with thickness of 3 mm were subjected to thermal performance and tensile strength tests, whereas films with thickness of 500 μm were used to study the DC conductivity and space charge distribution.

Additional pure XLPE samples and 0.007 wt% graphene-XLPE samples were prepared for water tree aging test. The thickness of the samples was 3 mm. On the surface of each sample, a 25 mm diameter circular area was chosen as the active aging zone, among which 18 pinholes with depths of 1.5 mm and tip radius of 4.0 ± 0.5 μm were embedded as the initiation spots of the water trees.

### Thermal characterization

Thermal performance of the samples was studied by using a Mettler-Toledo differential scanning calorimeter (DSC). Before the measurement, the samples were thermal-erased first, and then heated to 200 °C at a rate of 10 °C/min, during which the heat flow was recorded. Based on the result, crystallinity degree and crosslinking degree of the samples were calculated.

### Tensile strength measurement

Tensile strength of graphene/XLPE samples were investigated under the guidance of ISO 527-2012. The sample was first cut into a dumbbell shape and then subjected to tensile test on an elongation tester (TianFa Inc., JDL-5000 N), at a strain rate of 30 mm/min.

### DC conductivity measurement

DC conductivities of the pure XLPE sample and graphene/XLPE samples were measured by using a three-electrode structure with a Keithley 6517B connected in series. The measurement was performed strictly in accordance with IEC 62631, under 2 kV/mm, and 25 °C, 40 °C, 50 °C, 60 °C, 70 °C, respectively.

### Space charge measurement

The space charge distribution was investigated by the pulsed electro acoustic (PEA) method under room temperature 25 °C^43^. Samples of 500 μm in thickness and 10 cm in diameter were sandwiched between an aluminum electrode and a semiconductive polymer electrode. As the measurement started, a DC electric field of 30 kV/mm was applied to the sample and kept for 30 min. Afterwards, the depolarization current of the sample was recorded for another 1000 s.

### Water tree test

**Figure 7:**
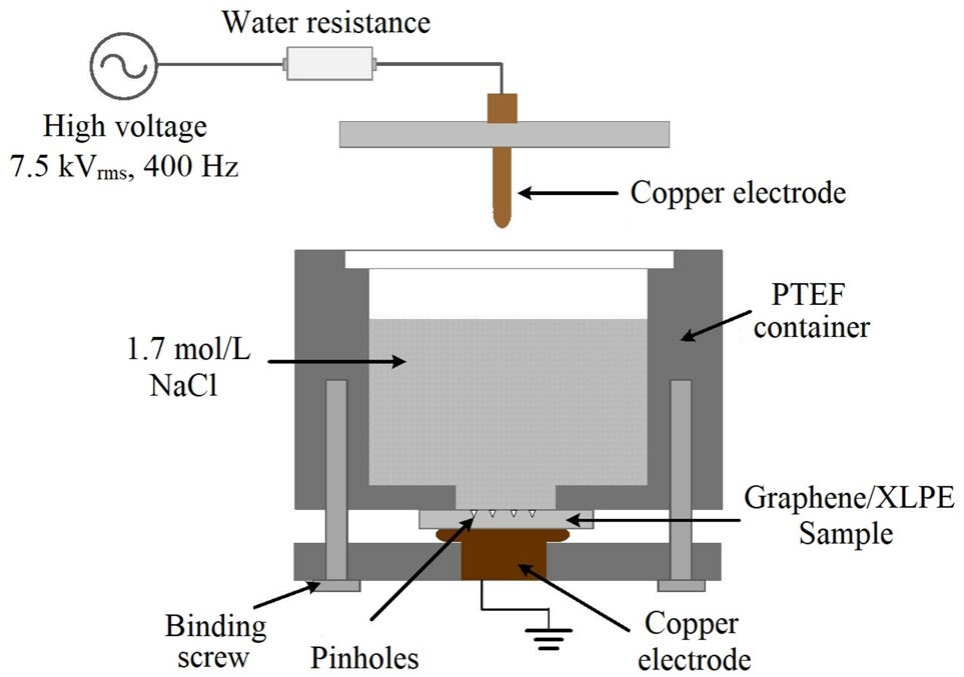
Experimental platform of the water tree aging test.

As for the water tree test, experimental platform shown in Fig. 7 was adopted.

The platform mainly consists of two parts: I. high voltage generating part, and II. water tree aging part. In part I, a signal generator in series with a power amplifier was adopted to generate 7.5 kVrms voltage with a frequency of 400 Hz. The high voltage was then applied to a rod copper electrode through a water resistance (≈ 10 kΩ). In part II, the 0.007 wt% graphene/XLPE sample with a thickness of 3 mm was sandwiched by a PTEF container filled with 1.7 mol/L NaCl solution, and a grounded copper electrode. Note that a 35 mm diameter circular area was hallowed at the bottom of the container, so that NaCl solution can directly immerse the pinholes on the sample. To transmit the high voltage to the pinhole tips of the graphene/XLPE sample, the rod copper electrode was inserted into the NaCl solution. Under the co-influence of moisture and electrical stress, water trees were therefore generated at the pinhole tips of the sample.
